# Toward a Fully Analytical Contact Resistance Expression in Organic Transistors

**DOI:** 10.3390/ma12071169

**Published:** 2019-04-10

**Authors:** Chang-Hyun Kim, Gilles Horowitz

**Affiliations:** 1 Department of Electronic Engineering, Gachon University, Seongnam 13120, Korea; 2 LPICM, Ecole Polytechnique, CNRS, 91128 Palaiseau, France

**Keywords:** organic field-effect transistors, contact resistance, device physics

## Abstract

Contact resistance is a major characteristic of organic transistors, and its importance has received renewed attention due to the recent revelation of mobility overestimation. In this article, we propose a method to describe the contact resistance as a closed-form compact equation of the materials, interfaces, and geometrical parameters. The proposed model allows us to quantitatively understand the correlation between charge-injection and transport properties, while providing a tool for performance prediction and optimization. This theory is applied to a set of experimentally fabricated devices to exemplify how to utilize the model in practice.

## 1. Introduction

Clearly, the organic field-effect transistor (OFET) has experienced a tremendous progress over its now more than three decades of history [1]. The initially widespread concerns about stability and reliability have been largely overcome, and the device performance has increased so much that the OFET technologies could successfully demonstrate the applicability to flat-panel displays [2], active-matrix imagers [3], radio-frequency identification tags [4], and many other areas. However, relatively immature understanding of the basic operation of OFETs limits sustainable developments. A lack of holistic understanding directly translates into a lack of dedicated compact model, which otherwise is critical to circuit simulation [5]. In other words, there is still no standard OFET model in general-purpose simulators, and often one is obliged to go with a Si transistor model available in the library. Also, when an OFET is used as a test bed for material inspection, there is sometimes no better choice than picking up the simplistic current-voltage equation that is for an ideal field-effect transistor. Since the latter usage of a ‘model’ is broadly evoked, there has been much debate on whether the common practice of charge-carrier mobility (*μ*) extraction should be revised [6,7,8,9].

At the heart of these parameterization issues is the contact resistance (*R_c_*) and its impact on the overall device behaviors. If we ‘assume’ that there is no parasitic *R_c_* and the channel resistance (*R*_ch_, which is a direct indicator of *μ*) is solely responsible for a measured drain current (*I_D_*), it is easy to underestimate the real *μ* when there is a non-zero *R_c_*, because we have got a higher *R*_ch_ than real [10]. Nonetheless, as explained by Liu and co-workers [11], both resistive components can be gate-voltage (*V_G_*) dependent to different extents, and we may either under- or overestimate the actual *μ* depending on the relative strength of the *V_G_* derivatives of these two resistances.

Completely eliminating *R_c_* in a device might be the best way to produce a high current and avoid analysis problems, but in most case, especially with a high-mobility material and/or at a large *V_G_*, this task is not straightforward. Therefore, it is recommended to directly measure the *V_G_*-dependent *R_c_* by e.g. transmission-line method (TLM) [12], four-probe measurement [13], or surface potentiometry [14], and then unambiguously deduce *R*_ch_ and *μ*. In parallel, deeply understanding what makes up *R_c_* and capturing this knowledge into a widely valid parametric form is of fundamental importance, as such a model allows us to see one step further and discuss the extracted *R_c_* and its implications.

In this article, we introduce a method to simplify the carrier distribution at the on-state of an OFET to build a fully analytical *R_c_* model. The model is based on the idea of effective accumulation thickness and it provides a direct link between *R_c_*and other key materials and device parameters, including *μ*. Therefore, it allows for the self-consistent decoupling and correlation between carrier injection and transport quantities, and provides a useful instrument for physical analysis.

## 2. Theoretical Modeling

An in-depth foundation of the following approach is given in our previous paper [15]. That study proposed the first theoretical model for the *R_c_* in coplanar OFETs, by defining the sharp carrier-density-transition zone at the source/channel interface as the responsible volume and expressing the single effective macroscopic resistance therein. While it convincingly demonstrated both the analytic and predictive capability of such a model, a major drawback was that it suggested a ‘semi-analytical’ final equation in that solving for *R_c_* involves numerical integration.

**Figure 1:**
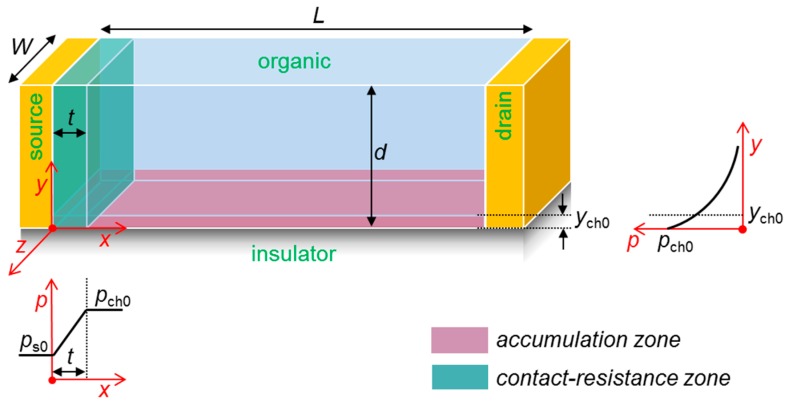
The conceptual representation of the 3-D organic channel in an organic field-effect transistor (OFET) that we employed for developing a contact resistance (*R_c_*) model (not to scale). A bottom-gate configuration is assumed, and the gate electrode is not shown for simplicity. All physical parameters are defined in the main text.

Therefore, we propose here a method to modify the theory toward a fully analytical and widely applicable compact model. We first summarize the major characteristics of the previous model using Figure 1, which visualizes the channel region of a p-type OFET. Here, *W* and *L* denote the channel width and length, respectively, and *d* is the semiconductor film thickness. The *x*, *y*, and *z* axes are defined as shown. Note that the model in [15] was originally dedicated to coplanar OFETs, but we believe that it can be a good approximation to staggered OFETs with a thin active layer where metal diffusion can shape vertically penetrating metal/organic interfaces [16].

From the Boltzmann statistics, the source hole density at *y* = 0 (*p_s_*_0_) is injection-limited, written as

(1) $$p_{s0}=N_{v}\exp\left(-\frac{E_{b}}{kT}\right),$$

where *N_v_* is the effective density of states (DOS) at the highest-occupied molecular orbital (HOMO) edge, *E_b_* is the hole injection barrier, *k* is the Boltzmann constant, and *T* is the absolute temperature. In contrast, the channel hole density at *y* = 0 (*p*_ch0_) is capacitively modulated. The widely used charge per area versus *V_G_* equation can be transformed to the carrier density dimension by assuming a strong vertical localization, as

(2) $$p_{\mathrm{ch}0}=\frac{C_{i}^{2}{\left(V_{G}-V_{T}\right)}^{2}}{2\varepsilon_{s}kT},$$

where *C_i_* is the insulator capacitance per area, *V_T_* is the threshold voltage, and *ε_s_* is the semiconductor permittivity. As illustrated in Figure 1, *p*_s0_ and *p*_ch0_ are independently controlled by Equations (1) and (2) and they fix a boundary condition of the respective carrier distribution, which decreases as *y* increases. In [15], we formulated these distributions as *p_s_*(*y*) and *p*_ch_(*y*). The key finding was an orders-of-magnitude difference between *p_s_* and *p*_ch_ values under realistic conditions (*p*_ch_ ≫ *p_s_*), rationalizing the emergence of *R_c_* at the source edge (a low carrier density means a high resistance). We therefore designated this transition zone with the length *t* as the physical *R_c_* region. The parameter *t* basically is a balance factor that shows how large such a zone should be to regulate the horizontal relaxations of *p_s_* and *p*_ch_, and has the following dependence.

(3) $$t=x_{s}\ln\left(\frac{p_{\mathrm{ch}}}{p_{s}}\right),$$

where *x_s_* is the Debye length of source carriers. The average hole density *p_m_* at this zone is given as

(4) $$p_{m}=\frac{1}{t}\int_{0}^{t}p\left(x\right)dx≅\frac{p_{\mathrm{ch}}}{\ln\left(p_{\mathrm{ch}}/p_{s}\right)}.$$

Therefore, the numerically integrated contact resistance, which we call *R*_num_ here, is obtained as

(5) $$R_{\mathrm{num}}\left(d\right)={\left[\int_{0}^{d}q\mu p_{m}\left(y\right)\frac{W}{t\left(y\right)}dy\right]}^{-1}.$$

Note here that *R_c_* does have a direct dependence on *μ*. Equation (5) can be supplemented by a specific functional form of *μ* such as

(6) $$\mu =\kappa {\left|V_{G}-V_{T}\right|}^{\alpha },$$

which assumes a charge transport mediated by an exponential trap DOS [5]. Here, *κ* is the mobility pre-factor and *α* is the characteristic exponent.

**Figure 2:**
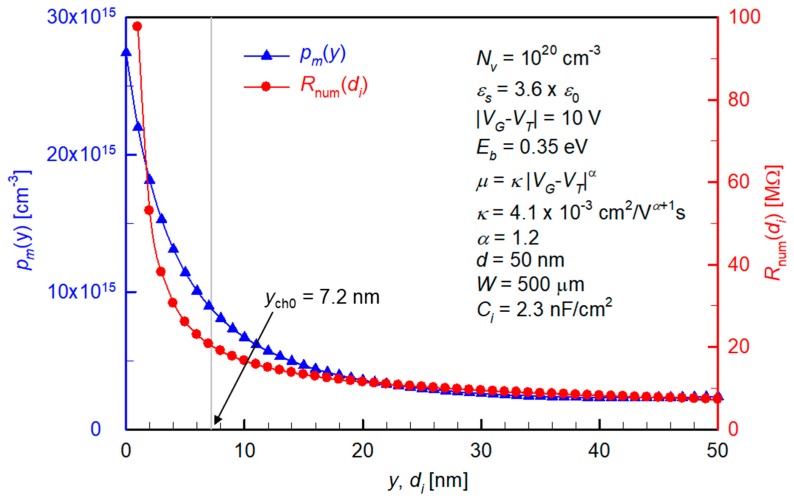
Calculation results showing the film-thickness-direction variations of *p_m_* (blue triangles) and *R*_num_ (red circles) under a fixed set of parameters. These fixed parameters are written on the graph (*T* = 300 K).

Figure 2 shows the change of *p_m_* with *y* and the numerically calculated *R*_num_ with a varying upper limit of integral *d_i_*, under fixed materials, geometrical, and bias conditions. These results suggest the two important keys to the modeling.

(1) Here, both *p_m_* and *R*_num_ decrease rapidly when moving from the insulator (*y*, *d_i_* = 0) to the top surface of the semiconductor (*y*, *d_i_* = 50 nm). Therefore, we choose to consider only the region 0 < *y* < *y*_ch0_, with the *y*_ch0_ being the effective accumulation thickness from the Mott–Gurney distribution. The value of *y*_ch0_ therefore decreases when *V_G_* increases (in magnitude) showing a smaller geometrical spread of the carriers at a higher gate field.
(2) In order to replace an integral with a simpler multiplication, we now need to deal with those that change with *y* in Equation (5), namely *p*_m_(*y*) and *t*(*y*). There are different ways to do this. For maximum simplicity, we take *p_m_*_0_ and *t*_0_ as the representative values that are determined by *p_s_*_0_ and *p*_ch0_, and are constant over 0 < *y* < *y*_ch0_. Note that this choice is also justified by the fact that while *p_m_*_0_ is the maximum of *p_m_*(*y*) at an actual distribution (e.g., Figure 2), assuming a constantly high *p_m_*_0_ over a certain physical distance can partly compensate the holes outside the effective accumulation space (i.e., those that are at *y* > *y*_ch0_)

Finally, the new modified *R_c_* model is constructed from

(7) $$R_{c}=\frac{t_{0}}{q\mu p_{m0}Wy_{\mathrm{ch}0}},$$

where

(8) $$t_{0}=x_{s0}\ln\left(\frac{p_{\mathrm{ch}0}}{p_{s0}}\right),$$

(9) $$p_{m0}=\frac{p_{\mathrm{ch}0}}{\ln\left(p_{\mathrm{ch}0}/p_{s0}\right)},$$

(10) $$y_{\mathrm{ch}0}=\sqrt{\frac{2\varepsilon_{s}kT}{q^{2}p_{\mathrm{ch}0}}},$$

Putting Equations (1), (2), (8)–(10), and $x_{s0}=\sqrt{\varepsilon_{s}kT/q^{2}p_{s0}}$ into Equation (7), this equation can be re-written as

(11) $$R_{c}=\frac{{\left[\ln\frac{C_{i}^{2}{\left(V_{G}-V_{T}\right)}^{2}}{2\varepsilon_{s}kTN_{v}}+\frac{E_{b}}{kT}\right]}^{2}}{q\mu W\sqrt{N_{v}}\exp\left(-\frac{E_{b}}{2kT}\right)\frac{C_{i}\left|V_{G}-V_{T}\right|}{\sqrt{\varepsilon_{s}kT}}},$$

which is a fully analytical model with explicit parametric impacts on the value of *R_c_*. We additionally note that retaining the absolute sign in |*V_G_* − *V_T_*| generalizes the model to both n- and p-type OFETs, with an obvious need to replace *N_v_* and *E_b_* by values associated with the lowest-unoccupied molecular orbital (LUMO) and the electron injection when applying it to an n-type device.

## 3. Application and Analysis

Major advantages of a closed-form analytical equation include an ease of application to practical research and a reduced computational load in circuit simulation. For instance, Equation (11) does not only illustrate the widely observed *V_G_* and *E_b_* dependence of *R_c_*, but it also clearly and quantitatively demonstrates a considerable impact of e.g. *ε_s_*and *C_i_*, which have been less considered. Therefore, such a model allows for assessing critical parameters and setting a strategy for rational optimization.

Here, we briefly describe how to apply the model to fabricated devices, in this case to estimate the *E_b_*. We repeat that it is crucial to robustly decouple contact and channel properties by systematic measurements before proceeding to any further analyses, to avoid parameter ambiguities [7,14]. We conducted the TLM on the pentacene OFETs with Au source/drain electrodes, whose fabrication and characterization details can be found in [15]. Note that full utilization of TLM gives access to both *R_c_* and (intrinsic) *μ* as a function of *V_G_* for a given set of length-variable transistors [12].

**Figure 3:**
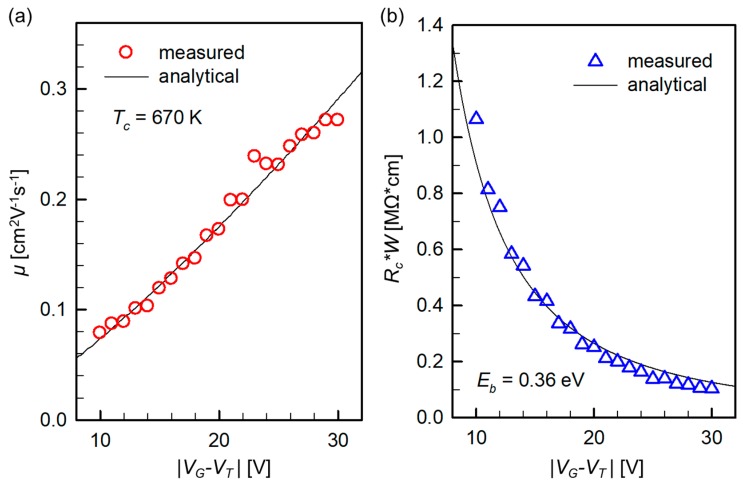
(**a**) *V_G_*-dependent *μ* in experimental devices overlaid with an analytical power-law mobility model. (**b**) Comparison between an experimentally measured and the model-produced *R_c_* versus *V_G_* trend, leading to an estimation of *E_b_*.

In Equation (11), we left the term *μ* intact, in view of possibly diverse transport scenarios and corresponding models. Once we have experimentally obtained a *μ* versus *V_G_* plot, it can be a perfect moment to check which model suits best the given devices. As shown in Figure 3a, our pentacene OFETs exhibit a substantial gate-field enhancement, so we fit the measured curve to the power-law model in Equation (6). A satisfactory fit was obtained, from which we extracted the characteristic temperature for the trap distribution *T_c_* [17,18] as 670 K by

*T_c_* = *T*(*α* + 1) (12)

Then, we inserted Equation (6) into Equation (11), assumed *N_v_* and *ε_s_*as those in Figure 2 [19], and fit the TLM-measured *R_c_* versus *V_G_* curve to Equation (11). Figure 3b shows that the model is in good agreement with the measurements, and the fitting process resulted in *E_b_* = 0.36 eV, which is a realistic value for a pentacene/Au junction [20].

As a final note, we clarify that the Boltzmann approximation for Equation (1) is broadly valid in organic devices where a large HOMO-LUMO gap and trap-related level pinning sustains a sizable energetic distance between the transport orbital and the electrode Fermi level [21]. If we incorporate the Fermi–Dirac distribution into our model despite this fact, its analytical simplicity will be reduced, but the predicted *R_c_* values will remain similar because of a sufficiently large *E_b_*.

## 4. Conclusions

In this article, we have proposed the strategic modification of the existing charge-based model toward a new fully analytical *R_c_* model. In addition to providing a convenient tool for OFET contact analysis and engineering, we believe that the systematic approach introduced here will inspire the physical and mathematical modeling of other key characteristics of OFETs, which will be ultimately necessary for a universal compact model of organic transistor technologies.

## References

[1] Tsumura A., Koezuka H., Ando T. (1986). Macromolecular electronic device: Field-effect transistor with a polythiophene thin film. Appl. Phys. Lett..

[2] Steudel S., Myny K., Schols S., Vicca P., Smout S., Tripathi A., van der Putten B., van der Steen J.-L., van Neer M., Schütze F. (2012). Design and realization of a flexible QQVGA AMOLED display with organic TFTs. Org. Electron..

[3] Nausieda I., Ryu K., Kymissis I., Akinwande A.I., Bulovic V., Sodini C.G. (2008). An organic active-matrix imager. IEEE Trans. Electron. Devices.

[4] Fiore V., Battiato P., Abdinia S., Jacobs S., Chartier I., Coppard R., Klink G., Cantatore E., Ragonese E., Palmisano G. (2015). An integrated 13.56-MHz RFID tag in a printed organic complementary TFT technology on flexible substrate. IEEE Trans. Circuits Syst. I-Regul. Pap..

[5] Kim C.-H., Bonnassieux Y., Horowitz G. (2014). Compact DC modeling of organic field-effect transistors: Review and perspectives. IEEE Trans. Electron. Devices.

[6] Choi D., Chu P.-H., McBride M., Reichmanis E. (2015). Best practices for reporting organic field effect transistor device performance. Chem. Mater..

[7] Bittle E.G., Basham J.I., Jackson T.N., Jurchescu O.D., Gundlach D.J. (2016). Mobility overestimation due to gated contacts in organic field-effect transistors. Nat. Commun..

[8] Choi H.H., Cho K., Frisbie C.D., Sirringhaus H., Podzorov V. (2017). Critical assessment of charge mobility extraction in FETs. Nat. Mater..

[9] Paterson A.F., Singh S., Fallon K.J., Hodsden T., Han Y., Schroeder B.C., Bronstein H., Heeney M., McCulloch I., Anthopoulos T.D. (2018). Recent progress in high-mobility organic transistors: A reality check. Adv. Mater..

[10] Horowitz G., Hajlaoui M.E. (2001). Grain size dependent mobility in polycrystalline organic field-effect transistors. Synth. Met..

[11] Liu C., Li G., Di Pietro R., Huang J., Noh Y.-Y., Liu X., Minari T. (2017). Device physics of contact issues for the overestimation and underestimation of carrier mobility in field-effect transistors. Phys. Rev. Appl..

[12] Kim C.-H., Hlaing H., Hong J.-A., Kim J.-H., Park Y., Payne M.M., Anthony J.E., Bonnassieux Y., Horowitz G., Kymissis I. (2015). Decoupling the effects of self-assembled monolayers on gold, silver, and copper organic transistor contacts. Adv. Mater. Interfaces.

[13] Hamai T., Arai S., Minemawari H., Inoue S., Kumai R., Hasegawa T. (2017). Tunneling and origin of large access resistance in layered-crystal organic transistors. Phys. Rev. Appl..

[14] Kim C.-H., Thomas S., Kim J.H., Elliott M., Macdonald J.E., Yoon M.-H. (2018). Potentiometric parameterization of dinaphtho[2,3-b:2’,3’-f]thieno[3,2-b]thiophene field-effect transistors with a varying degree of nonidealities. Adv. Electron. Mater..

[15] Kim C.H., Bonnassieux Y., Horowitz G. (2013). Charge distribution and contact resistance model for coplanar organic field-effect transistors. IEEE Trans. Electron Devices.

[16] Braga D., Horowitz G. (2009). High-performance organic field-effect transistors. Adv. Mater..

[17] Kim C.-H., Hlaing H., Payne M.M., Yager K.G., Bonnassieux Y., Horowitz G., Anthony J.E., Kymissis I. (2014). Strongly correlated alignment of fluorinated 5,11-bis(triethylgermylethynyl)anthradithiophene crystallites in solution-processed field-effect transistors. ChemPhysChem.

[18] Kalb W.L., Batlogg B. (2010). Calculating the trap density of states in organic field-effect transistors from experiment: A comparison of different methods. Phys. Rev. B.

[19] Kim C.H., Yaghmazadeh O., Tondelier D., Jeong Y.B., Bonnassieux Y., Horowitz G. (2011). Capacitive behavior of pentacene-based diodes: Quasistatic dielectric constant and dielectric strength. J. Appl. Phys..

[20] Hwang J., Wan A., Kahn A. (2009). Energetics of metal-organic interfaces: New experiments and assessment of the field. Mater. Sci. Eng. R-Rep..

[21] Kim C.-H., Kymissis I. (2017). Graphene–organic hybrid electronics. J. Mater. Chem. C.

